# Cohort Profile: Chinese Cervical Cancer Clinical Study

**DOI:** 10.3389/fonc.2021.690275

**Published:** 2021-06-18

**Authors:** Xi-Ru Zhang, Zhi-Qiang Li, Li-Xin Sun, Ping Liu, Zhi-Hao Li, Peng-Fei Li, Hong-Wei Zhao, Bi-Liang Chen, Mei Ji, Li Wang, Shan Kang, Jing-He Lang, Chen Mao, Chun-Lin Chen

**Affiliations:** ^1^ Department of Epidemiology, School of Public Health, Southern Medical University, Guangzhou, China; ^2^ Department of Obstetrics and Gynecology, Nanfang Hospital, Southern Medical University, Guangzhou, China; ^3^ Department of Gynecologic Oncology, Shanxi Provincial Cancer Hospital, Taiyuan, China; ^4^ Department of Gynecology, Yanling Hospital of Southern Medical University, Guangzhou, China; ^5^ Department of Obstetrics and Gynecology, Xijing Hospital of Airforce Medical University, Xi’an, China; ^6^ Department of Obstetrics and Gynecology, The First Affiliated Hospital of Zhengzhou University, Zhengzhou, China; ^7^ Department of Gynecologic Oncology of Affiliated Cancer Hospital of Zhengzhou University, Zhengzhou, China; ^8^ Department of Gynecology, The Forth Hospital of Hebei Medical University, Shijiazhuang, China; ^9^ Department of Obstetrics and Gynecology, Peking Union Medical College Hospital, Peking Union Medical College, Beijing, China

**Keywords:** cervical cancer, cohort, therapy, prognosis, prediction

## Abstract

Cervical cancer is the fourth most common cancer worldwide, but its incidence varies greatly in different countries. Regardless of incidence or mortality, the burden of cervical cancer in China accounts for approximately 18% of the global burden. The Chinese Cervical Cancer Clinical Study is a hospital-based multicenter open cohort. The major aims of this study include (i) to explore the associations of therapeutic strategies with complications as well as mid- and long-term clinical outcomes; (ii) to widely assess the factors which may have an influence on the prognosis of cervical cancer and then guide the treatment options, and to estimate prognosis using a prediction model for precise post-treatment care and follow-up; (iii) to develop a knowledge base of cervical clinical auxiliary diagnosis and prognosis prediction using artificial intelligence and machine learning approaches; and (iv) to roughly map the burden of cervical cancer in different districts and monitoring the trend in incidence of cervical cancer to potentially inform prevention and control strategies. Patients eligible for inclusion were those diagnosed with cervical cancer, whether during an outpatient visit or hospital admission, at 47 different types of medical institutions in 19 cities of 11 provinces across mainland China between 2004 and 2018. In a total, 63 926 patients with cervical cancer were enrolled in the cohort. Since the project inception, a large number of standardized variables have been collected, including epidemiological characteristics, cervical cancer-related symptoms, physical examination results, laboratory testing results, imaging reports, tumor biomarkers, tumor staging, tumor characteristics, comorbidities, co-infections, treatment and short-term complications. Follow-up was performed at least once every 6 months within the first 5 years after receiving treatment and then annually thereafter. At present, we are developing a cervical cancer imaging database containing Dicom files with data of computed tomography/magnetic resonance imaging examination. Additionally, we are also collecting original pathological specimens of patients with cervical cancer. Potential collaborators are welcomed to contact the corresponding authors, and anyone can submit at least one specific study proposal describing the background, objectives and methods of the study.

## Highlights

- Starting in 2014, we developed the Chinese Cervical Cancer Clinical Study, a hospital-based open cohort, to assess outcomes of different management strategies on cervical cancers with specific clinical stages, and evaluate the influence of various prognostic factors on the oncological outcomes to guide treatment options, care, and follow-up.
- Up to now, this cohort has recruited 63 926 patients with cervical cancer (mean [SD] age, 49.19 [10.44] years) from 47 general, cancer, as well as women’s and children’s hospitals distributed in 26 cities of 14 provinces across the mainland China.
- A total of 315 standardized variables were collected by reviewing the patients’ medical records, which covered almost all information on epidemical characteristics, clinical testing results, examinations clinical diagnoses, treatments and prognosis at baseline. Follow-up was conducted at certain time intervals.
- We are building a cervical cancer imaging database to collect the Dicom file data of computed tomography and/or magnetic resonance imaging examinations. Additionally, we are collecting original pathological samples of patients with cervical cancer. Based on those, we might conduct extensive researches on imaging omics and pathology omics of cervical cancer using artificial intelligence, machine learning and advanced biological technology.

## Why Was the Cohort Set up?

Cervical cancer is a major public health problem. It is the fourth most common cancer type in terms of incidence and mortality in women worldwide. In 2018, an estimated 570 000 cases of cervical cancer and 311 000 deaths caused by cervical cancer have been recorded (1–5). The incidence and mortality rates of cervical cancer varied widely among countries (1). With the highest number of cases (106 430) and the second-highest estimated number of deaths (47 739), China accounts for approximately 18% of the global cervical cancer burden (6).

The elimination of cervical cancer as a public health issue is considered a priority under the WHO 13th General Programmer of Work of the World Health Organization (7–9). Despite the wide availability of screening and improved therapeutic practices, the 5-year overall survival (OS) of cervical cancer remains at only 60–70% in high-income countries and it is much lower in middle- and low-income countries (10). Therefore, more reasonable prevention and control strategies must be developed. And more effective diagnosis and treatment strategies must be applied in clinical practice. These strategies are associated with a shorter potential years of life lost and a fewer disability-adjusted life year lost due to cervical cancer (11).

The effect of different management on patients with specific clinical stages of cervical cancer remains controversial (12–15). Prognostic factors, including tumor characteristics, medical condition, sociodemographic characteristics, lifestyle behaviors, biomarkers, diagnosis, treatment, and care, are suggested for further detailed exploration (16–22). This may provide a potential way to reduce residual disease after treatment, as well as minimize recurrence, decrease the complications, and improve survival. Furthermore, the recent advances in medical information technology and computer technology play an important role in the realization of auxiliary diagnosis and digital healthcare of cervical cancer (23, 24). These developments provide an optimism outlook for remarkable progress in the diagnosis, treatment, and prediction of the prognosis of cervical cancer.

The Chinese Cervical Cancer Clinical (Four-C) Study was created in 2014 with the aim of collecting clinical and prognostic information on patients diagnosed with cervical cancer in mainland China since 2004. Its research objectives currently focus on four main themes: (i) to explore the associations of therapeutic strategies with complications as well as mid- and long-term clinical outcomes, including comparative effectiveness research based on marginal structural models or propensity scores (25–27); (ii) to widely evaluate the prognostic factors of cervical cancer (such as late access to care and the influence of nutritional status) and then guide treatment as well as care options, and to precisely predict the prognosis of patients so as to develop much more effective program of personalized follow-up and intervention (21, 22); (iii) to utilize artificial intelligence (AI) and machine learning (ML) approaches for multimodal data aggregation and multifactorial examination in order to develop a knowledge base of cervical clinical auxiliary diagnosis and prognostic prediction (28–30). What’ more, as the Four-C Study relatively represents the occurrence of cervical cancer across mainland China in terms of age, geographical origin, year of diagnosis, clinical stage, gross type, and histological type, it can also serve to map the burden of cervical cancer in different districts and monitor trends in incidence of cervical cancer, which could potentially inform prevention and control strategies (31).

## Who Is in the Cohort?

**Figure 1:**
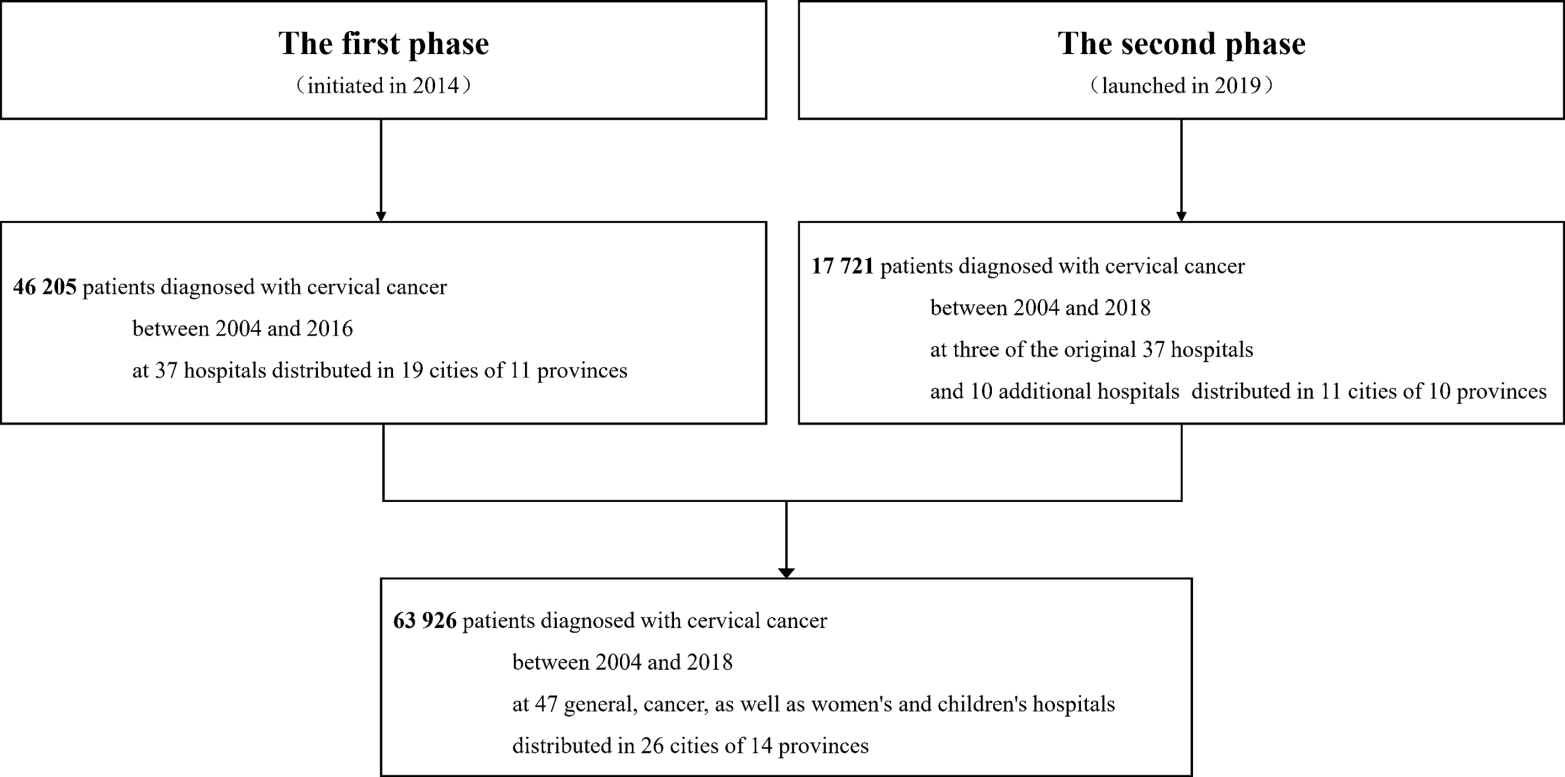
Flowchart of participant enrolment.

**Figure 2:**
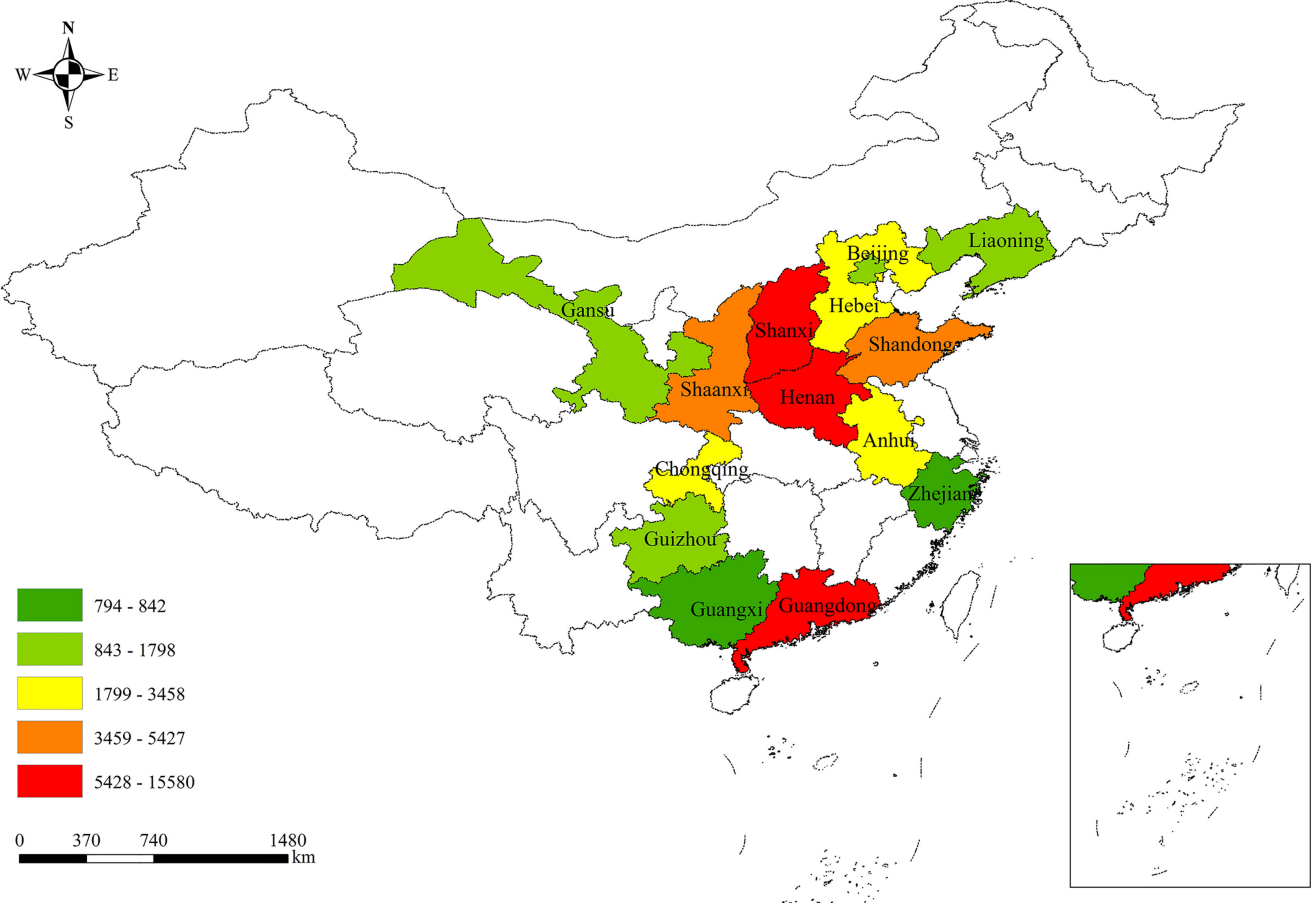
The province distribution of the study participants.

The Four-C Study was set up by two phases. The inclusion criteria for the participants were as follows: subjects who were outpatients or inpatients of participating centers of the Four-C Study; subjects who have a pathology report of cervical biopsy, which is the gold standard for cervical cancer diagnosis, issued by at least two experienced doctors in a Grade III Level A hospital. In the first phase initiated in 2014, the hospital-based open cohort recruited 46 205 patients diagnosed with cervical cancer between 2004 and 2016 at 37 hospitals distributed in 19 cities of 11 provinces across mainland China. The second phase was launched in 2019. Three of the original 37 hospitals (Guizhou Provincial People’s Hospital, Shanxi Provincial Cancer Hospital, and The Yuncheng Central Hospital) continuously participated in the second phase. Additionally, other 10 hospitals collected epidemiological and clinical data of 17 721 patients who was diagnosed with cervical cancer between 2004 and 2018. In a total, 63 926 patients with cervical cancer from 47 general hospitals, cancer hospitals, as well as women’s and children’s hospitals distributed in 26 cities of 14 provinces across the mainland China were enrolled in the large-scale open subject cohort (**Figure 1**). The geographical distribution of all the participants is shown in **Figure 2**.

The documentation of patient information included epidemical characteristics, clinical testing results, examinations, diagnoses, treatments, care and prognosis. Further details on the project are available at the International Clinical Trails Registry Platform (http://apps.who.int/trialsearch/, ChiCTR1800017778). Approval was obtained from the institutional ethics committee of Nanfang Hospital (NFEC-2017-135) (32, 33). Only the information on the medical practice of each patient was collected so that individual patients could not be identified. The ethics committee exempted informed consent. Of note, every patient is assigned a unique number to match the clinical/epidemiological information obtained from medical records with original pathology specimens or image data, which can be used to validate a diagnosis or provide additional information for subsequent specific research projects.

## What Has Been Measured and Collected?

At baseline, the Four-C Study collected 315 epidemiological and clinical variables by returning to the patients’ medical records, which covered almost all information on clinical diagnosis, treatment, care and short-term outcome of cervical cancer, including sociodemographic characteristics, menstrual history and reproductive history, type of medical institution, cervical cancer-related symptoms, physical examination, laboratory testing, imaging report, tumor biomarkers, tumor staging, tumor characteristics, comorbidities, co-infections, management of patients and short-term complications after treatments. **Table 1** shows a broad overview of the information collected on each inclusion participant. Further detailed information on management of patients, such as surgical approaches, surgical procedures, other surgical-related information, and the complete protocols radiotherapy or chemotherapy, were also documented, which was summarized in **Table 2**. The tumor staging was defined by the International Federation of Gynecology and Obstetrics (FIGO) staging system in 2009 and 2018.

**Table 1:** Epidemiological and clinical data in the Chinese Cervical Cancer Clinical Study.

Components	Measurements
Baseline data	
Sociodemographic characteristics	date of birth, age at first diagnosis, ethnicity, region, Province, City, city scale, education level, occupation, residence, marital status, age of marriage, sexual life and family history of cervical cancer
Menstrual history and reproductive history	age of menarche, pregnancy history, age of childbearing, parity, delivery way and hormone replacement therapy history
Type of medical institution	general hospital, cancer center, women and children’s center
Related symptoms	anemia, leukorrhagia, irregular vaginal bleeding, contact bleeding, a foul-smelling watery or sometimes bloody vaginal discharge, lower extremity edema, fever, oliguria or osphyalgia
Physical examination	bimanual pelvic examination, colposcopy, biopsy, height, weight, resting blood pressure, temperature, pulse, heart rate, the administration of 12-lead electrocardiography (ECG), auscultation heart and lung, hearing acuity, regular examinations of otorhinolaryngology, the heart and blood vessels examination, respiratory system examination, nervous system examination and abdominal viscera examination, limbs and joints movements, liver function
Laboratory testing	thinprep cytologic test (TCT), human papillomavirus testing (HPV testing), pathology report
Imaging report	computed tomography (CT), magnetic resonance imaging (MRI), endovaginal/transrectal ultrasound (US) and/or positron emission tomography-computed tomography (PET-CT)
Tumor biomarkers	squamous cell carcinoma antigen (SCC-Ag), carcinoembryonic antigen (CEA), Human epididymis protein 4 (HE4), carbohydrate antigen-153 (CA-153), Carbohydrate antigen199 (CA-199), carbohydrate antigen-125 (CA-125), tissue polypeptide antigen (TPA), tumor necrosis factor alpha (TNF-α) and interleukin- 6 (IL-6)
Tumor characteristics	tumor size, tumor location, pathological tumor type, gross type, tumor volume, a maximum depth of cervical stromal invasion, a minimum thickness of uninvolved cervical stroma, extracervical tumor extension and LN involvement (number, size, location), clinically visible lesion, presence or absence of lymph vascular space involvement (LVSI), presence or absence of distant metastases, TNM classification
FIGO stage	Using the International Federation of Gynecology and Obstetrics (FIGO) staging system in 2009 and 2018
Comorbidities	malignancies, hypertension, CVD, diabetes, kidney disease, pulmonary disease, gallbladder disorders
Medical and medication history	Surgical, drugs taken for diabetes, hypertension, CVD, kidney disease, respiratory diseases and digestive disease and immunosuppressive drug
Co-infection	hepatitis virus, herpes simplex virus (HSV), treponema pallidum, human immunodeficiency virus (HIV) or other virus
Management of patients	surgical treatment, primary radiotherapy, primary chemoradiotherapy, adjuvant radiotherapy, adjuvant chemo(radio)therapy, neoadjuvant chemotherapy, Neoadjuvant brachytherapy, hospital stay
Short-term complications	vascular injury, bladder injury, ureteral injury, bowel injury, stomach injury obturator nerve injury
Follow-up data	
Middle- and long-term complications	rectovaginal fistula, vesicovaginal fistula, ureterovaginal fistula, ureteral fistula, lymphedema, bowel obstruction, pelvic hematoma, venous thromboembolism, hemorrhage, chylous leakage, radiation proctitis, vaginitis, vulvitis, and bone marrow suppression
Management	Reexamination, treatment strategies for complications, care, hospitalization and medication
Recurrence	time of recurrence, location of recurrence and treatment after recurrence
Drug use	Drugs taken for diabetes, hypertension, CVD, kidney disease, respiratory diseases or digestive disease
Death information	time of death, cause of death

LN, lymph node; CVD, cardiovascular disease.

**Table 2:** Detailed information on treatment.

Components		Measurements
Surgical treatment	Surgical approaches	abdominal, vaginal, laparoscopic or robot-assisted
	Surgical procedures	cone/loop resection, resection margins, presence or absence of positive resection margins, trachelectomy, type of hysterectomy, nerve-sparing radical surgery, presence or absence of ovaries and fallopian tubes, lymphadenectomy, LN dissection, presence or absence of vaginal cuff, and presence or absence of parametria
	Other information	preoperative care, preoperative workup, pretreatment surgical, anesthesia, surgical margins, surgical interventions, blood transfusion, intraoperative blood loss, operation period, postoperative care, postoperative complications, postoperative recurrence, the time of postoperative first exhaust and defecation, the time of postoperative catheter removal, postoperative residual urine, residual disease, residual tumor or reoperation,
Radiotherapy	type of radiation source, radiation approach, area exposed, site exposed, unit dose, total dose, total number of segments and total treatment time as well as the effectiveness of radiotherapy	
Chemotherapy	drug, dose, course, interval and route	

LN, lymph node.

The distribution of baseline characteristics was displayed as the number (percentage [%]) for categorical variables and mean (standard deviation [SD]) for continuous variables (**Table 3**). Of the 63 926 patients (mean [SD] age, 49.19 [10.44] years), more than one-half (58.9%) lived in rural areas, approximately 70% lived in a second-tier city and 95.4% were married. When giving birth, vaginal delivery (79.6%) was the most common delivery method. Patients were most likely to receive treatment in a general hospital (31 997 cases, 50.1%), followed by a cancer center (29 893, 46.8%). There are 49852 (78.0%) patients with FIGO stage I–II, 5663 patients (8.9%) with FIGO stage III and 689 patients (1.1%) with FIGO stage VI. The gross tumor type was mainly exogenous (41.7%), and the pathological tumor type was generally squamous cell carcinoma (87.8%) and adenocarcinoma (6.9%). Of the 48962 patients who underwent surgery, abdominal hysterectomy was reported for 28482 cases (58.2%), laparoscopic surgery for 15499 (31.7%), robot-assisted hysterectomy for 2176 cases (4.4%) and vaginal hysterectomy for 628 (1.3%).

**Table 3:** Selected characteristics of participants in the Chinese Cervical Cancer Clinical Study.

Characteristics	Overall (N = 63 926)	The First stage (N = 46 205)	The second stage (N=17 721)	
			RRH Data (N = 2790)	General data (N = 14 931)
Age at first diagnosis, years	49.19 (10.44)	49.34 (10.55)	48.12 (10.05)	48.93 (10.12)
Age at first diagnosis, years				
≤45	24413 (38.19)	17586 (38.06)	1117 (40.04)	5710 (38.25)
46-69	37060 (57.97)	26642 (57.66)	1636 (58.64)	8782 (58.82)
≥70	2190 (3.43)	1768 (3.83)	37 (1.33)	385 (2.58)
Missing	263 (0.41)	209 (0.45)	0	54 (0.36)
Year of diagnosis				
2004-2006	4662 (7.3)	3891 (8.4)	25 (0.9)	746 (5.0)
2007-2010	13685 (21.4)	11585 (25.1)	62 (2.2)	2038 (13.7)
2011-2014	24972 (39.1)	20886 (45.2)	650 (23.3)	3436 (23.0)
2015-2018	20599 (32.2)	9843 (21.3)	2053 (73.6)	8703 (58.3)
Marital status				
Married	61002 (95.43)	43757 (94.70)	2667 (95.59)	14578 (97.64)
Unmarried	306 (0.48)	226 (0.49)	23 (0.82)	57 (0.38)
Divorced	684 (1.07)	512 (1.11)	50 (1.79)	122 (0.82)
Widowed	671 (1.05)	529 (1.14)	39 (1.40)	103 (0.60)
Remarried	231 (0.36)	200 (0.43)	11 (0.39)	20 (0.13)
Unknown	1032 (1.61)	981 (2.12)	0	51 (0.34)
Residence				
Rural	37626 (58.86)	26791 (57.98)	1422 (51.00)	9413 (63.00)
Urban	18419 (28.81)	12782 (27.66)	1027 (36.80)	4610 (30.90)
Unknow	7881 (12.33)	6632 (14.35)	341 (12.20)	908 (6.10)
Region				
North	19653 (30.7)	18410 (39.8)	400 (14.3)	843 (5.60)
South	10889 (17.0)	10633 (23.0)	256 (9.2)	0
Central	11571 (18.1)	7092 (15.3)	306 (11.0)	4173 (27.9)
East	7305 (11.4)	4563 (9.9)	0	2742 (18.4)
Southwest	5865 (9.2)	5507 (11.9)	0	358 (2.4)
Northwest	6499 (10.2)	0	1828 (65.5)	4671 (31.3)
Northeast	2144 (3.4)	0	0	2144 (14.4)
City scale				
First-tier	8948 (14.0)	8548 (18.5)	400 (14.3)	0
Second-tier	44929 (70.3)	29006 (62.8)	2310 (82.8)	13613 (91.2)
Third-tier and below	10049 (15.7)	8651 (18.7)	80 (2.90)	1318 (8.8)
Institution type				
General hospital	31997 (50.1)	16000 (34.6)	2534 (90.8)	13463 (90.2)
Cancer center	29893 (46.8)	28425 (61.5)	0	1468 (9.8)
Women and children center	2036 (3.2)	1780 (3.9)	256 (9.2)	0
Delivery types				
No delivery	624 (0.98)	529 (1.14)	38 (1.36)	57 (0.38)
Vaginal delivery	50864 (79.57)	35449 (76.72)	2332 (83.58)	13083 (87.62)
Cesarean delivery	2669 (4.18)	1643 (3.56)	229 (8.21)	797 (5.34)
Vaginal and cesarean delivery	822 (1.29)	524 (1.13)	71 (2.54)	227 (1.52)
Unknown	8947 (14.00)	8060 (17.44)	120 (4.30)	767 (5.14)
FIGO stage (2009)				
IA1	2496 (3.90)	1657 (1.07)	105 (3.76)	734 (4.92)
IA2	714 (1.12)	496 (27.83)	42 (1.51)	176 (1.18)
IB1	18036 (28.21)	12858 (7.92)	1182 (42.37)	3996 (26.76)
IB2	4716 (7.38)	3660 (1.54)	231 (8.28)	825 (5.53)
IA	1056 (1.65)	713 (1.26)	36 (1.28)	307 (2.06)
IB	928 (1.45)	581 (11.86)	30 (1.08)	317 (2.12)
IIA1	7702 (12.05)	5479 (6.40)	471 (16.88)	1752 (11.73)
IIA2	3901 (6.10)	2956 (2.32)	211 (7.56)	734 (4.92)
IIA	1455 (2.28)	1071 (12.40)	26 (0.93)	358 (2.40)
IIB	8542 (13.36)	5729 (1.25)	302 (10.82)	2511 (16.82)
IIIA	772 (1.21)	576 (8.30)	17 (0.61)	179 (1.20)
IIIB	4680 (7.32)	3833 (0.28)	25 (0.90)	822 (5.51)
I	177 (0.28)	129 (0.23)	0	48 (0.32)
II	129 (0.20)	108 (0.41)	3 (0.11)	18 (0.12)
III	211 (0.33)	188 (0.25)	1 (0.04)	22 (0.15)
IVA	145 (0.23)	115 (0.49)	2 (0.07)	28 (0.19)
IVB	241 (0.38)	228 (0.47)	1 (0.04)	12 (1.08)
IV	303 (0.47)	217 (1.38)	0	86 (0.58)
CIN	987 (1.54)	637 (10.77)	7 (0.25)	343 (2.30)
Unknown	6735 (10.54)	4974 (10.54)	98 (3.51)	1663 (11.14)
Gross type				
Erosion	809 (1.3)	809 (1.8)	0	0
Exophytic	26630 (41.7)	19154 (41.5)	1479 (53.0)	5997 (40.2)
Endophytic	5951 (9.3)	4993 (10.8)	98 (3.5)	860 (5.8)
ulcerative	7487 (11.7)	6686 (14.5)	115 (4.1)	686 (4.6)
Cervical canal	776 (1.2)	551 (1.2)	109 (3.9)	116 (0.8)
After conization	2047 (3.2)	1276 (2.8)	111 (4.0)	660 (4.4)
No found	16347 (25.6)	11130 (24.1)	785 (28.1)	4432 (29.7)
Unknown	3879 (6.1)	1606 (1.8)	93 (3.3)	2180 (14.6)
pathological tumor type				
Squamous cell carcinoma	56141 (87.8)	40612 (87.9)	2455 (88.0)	13074 (87.6)
Adenocarcinoma	4422 (6.9)	3037 (6.6)	243 (8.7)	1142 (7.8)
Adenosquamous carcinoma	992 (1.6)	757 (1.6)	43 (1.5)	192 (1.3)
Clear cell carcinoma	117 (0.2)	90 (0.2)	4 (0.1)	23 (0.2)
Small cell neuroendocrine carcinoma	372 (0.6)	279 (0.6)	1 (0.0)	92 (0.6)
Other subtypes	518 (0.8)	376 (0.8)	29 (1.0)	113 (0.8)
Unknown	1364 (2.1)	1054 (2.3)	15 (0.5)	295 (2.0)

FIGO, the International Federation of Gynecology and Obstetrics; CIN, cervical intraepithelial neoplasia.

## How Often Have They Been Followed up?

Follow-up was conducted by well-trained nursers, research assistants and postgraduates in Gynecology and Obstetrics through telephone conversations with the patient or one of her family members, or *via* the assessment of patient electronic medical notes (both at each outpatient visit or hospital admission) at certain time intervals defined by the follow-up strategy. The follow-up interval is every 3 to 4 months within the first 2 years, every 6 months for the next 3 years, and then annually thereafter or until year 10 at the discretion of the treating physician. Bedsides, detailed follow-up information of participants is require to be collected immediately if a special and meaningful clinical manifestation is identified or a significant change in tumor-related biological markers is noted during an outpatient visit for review after treatment.

Standardized variables were collected to evaluate the residual disease, residual tumor, surveillance, survival, and other information (**Table 1**). These included the time of follow-up, middle- and long-term complications, treatment strategies for complications, recurrence of cervical cancer and the time of recurrence, site of recurrence and treatment after recurrence, drug use, outpatient, hospitalization and death information.

We take some measures to minimize the loss of follow-up. Firstly, all hospitals have promised to strictly abide by the follow-up strategy. Upon discharge or departure from the clinic, each participant was notified in detail the time interval for re-examination and/or follow-up. The doctors in charge should maintain long-term communication with each patient to improve their compliance. Additionally, patients who move or seek care elsewhere were also traced, and information of those who died was collected through telephone conversations with one of her close family members.

## Quality Assurance and Control

First of all, after full discussion, preextraction and revision, a concise and standardized case report form (CRF) and a brief guideline for performing data extraction from the medical records were developed. Baseline information was abstracted from two systems: an electronic medical record (EMR) system and a conventional paper medical record kept in the hospital medical documents room, which ensured the integrity of data. Data were read and entered into electronic CRF by two trained gynecologists, nurses or postgraduates in obstetrics and gynecology, respectively. Subsequently, we checked the consistency of data for the same patient entered by different researchers and tried our efforts to correct any questionable data. Meanwhile, the information in the database was randomly examined by researchers designated by the study group. In this way, the accuracy and completeness of all the input information were guaranteed, even though it required significant labor and time costs. Finally, editing of the database was locked to prevent the modification or destruction of the determined data. Notably, all data were backed up at every stage of database development to prevent accidental loss of recorded data.

## What Has it Found? Key Findings and Publications

Although laparoscopic surgery and robot-assisted hysterectomy for cervical cancer have significantly increased since 2004, little is known about their real effect when applied in women in China with FIGO stage IA–IIB cervical cancer. In addition, the incidence of specific complications associated with radical hysterectomy, and the influence of prognostic factors (such as uterine corpus invasion, and urologic complication) on tumor outcomes remain unclear. This large hospital-based cohort duly contributed several studies to answer above questions based on evidence in the database.

### Higher Risk of Major Surgical Complications With Laparoscopic Radical Hysterectomy (LRH) Compared With Abdominal Radical Hysterectomy (ARH)

We compare a total of 18447 patients with FIGO stage IA–IIB cervical cancer diagnosed between 2004 and 2015 in China (34) to explore the rate of major complications associated with LRH (29.8%) and ARH (70.2%) for cervical cancer. We observed that the rate of LRH complications was 8.74% in 2009 and then was stabilized within the range of 4.80–6.56% between 2010 and 2015. The rate of ARH complications was relatively stable, with a range of 2.07–3.66%. Notably, our study showed that the LRH group had significant higher odds of intraoperative complications (OR = 3.88, 95% CI 2.47 to 6.11) and postoperative complications (OR = 1.42, 95% CI 1.11 to 1.82) compared with the ARH group. Furthermore, compared with ARH, LRH was related to an increased risk of urologic complications, such as intraoperative ureteral injury, bowel injury, postoperative vesicovaginal fistula, ureterovaginal fistula, rectovaginal fistula, and chylous leakage. The results suggest that LRH for cervical cancer may not be as minimally invasive as it seems.

### Urologic Complications With Radical Hysterectomy for Cervical Cancer Seemed to Have an Implicit on Short-Term But Not on Long-Term Survival

Of the 21 026 patients undergoing radical hysterectomy for early-stage cervical cancer (35), we documented 324 (1.54%) cases with at least one urologic complication. Variables significantly associated with a higher rate of urologic complications included being treated at a facility in a first-tier city (OR = 2.08, 95% CI 1.24 to 3.48), at a women’s and children’s hospital (OR = 2.26, 95% CI 1.47 to 3.48), and undergoing LRH (OR = 4.68, 95% CI 3.44 to 6.36). Furthermore, our study demonstrated that the occurrence of urologic complications was an independent risk factor for decreased 2-year overall survival (OS) (OR = 1.78, 95% CI 1.09 to 2.92), but not for decreased 5-year OS (OR = 1.27, 95% CI 0.83 to 1.94). Additionally, age ≥60, preoperative radiotherapy, FIGO stages IB2–IIB, positive pelvic lymph node, positive parametrial invasion, deep 1/2 stromal invasion, positive lymph vascular space (LVSI), tumor size >4 cm, and inadequate adjuvant treatment were also significant predictors of lower 5-year OS.

### Compared With ARH, Robot-Assisted Radical Hysterectomy (RRH) or LRH for Cervical Cancer Predicted Worse Oncological Outcomes

We published a study (36) that evaluated 3-year OS and disease-free survival (DFS) of RRH and ARH for patients with cervical cancer whose FIGO stages were IA1 with LVSI-IIA2. After propensity score matching, patients who underwent RRH were likely to have shorter rates of 3-year OS (94.4% *vs*. 97.8%, p = 0.002) and 3-year DFS (91.1% *vs*. 95.4%, p = 0.001) than those who underwent ARH. In multivariable models, RRH was associated with decreased rates of 3-year OS (HR = 2.86, 95% CI 1.59 to 5.16) and 3-year DFS (HR = 2.34, 95% CI 1.54 to 3.56). However, the ARH showed similar rates of 3-year oncological outcomes among patients with stage IB1 and tumor size <2 cm.

Another study (37) included 13413 participants with FIGO stage IA1 with LVSI-IIA2 cervical cancer. It showed that the rates of 5-year DFS of patients in the LRH group were lower than those in the ARH group (HR = 1.25, 95% CI 1.11 to 1.40). Compared with ARH, LRH was not an appropriate approach for patients whose FIGO stage was IB1 or IIA1 and tumor size was ≥2 cm. In other words, LRH or RRH could lead to worse oncological outcomes than ARH for patients with cervical cancer depending on the specific FIGO stage. These results demonstrate the need to be more cautious and considerate in the choice of surgical approaches.

### Uterine Corpus Invasion Should be Taken Seriously

Diagnosis of uterine corpus invasion is frequently missed. According to a retrospective review of original pathology specimens (38) there were 38 patients with a missed diagnosis of uterine corpus invasion and 20 patients were misdiagnosed with uterine corpus invasion among 1414 patients with FIGO stage IA2–IIB cervical cancer from 11 medical institutions in mainland China. We found that myometrial invasion ≥50% seemed to be an independent prognostic factor for decreased rates of 5-year OS (HR = 2.74, 95% CI 1.81 to 4.13) and 5-year DFS (HR = 2.31, 95% CI 1.59 to 3.35), whereas myometrial invasion <50% or endometrial invasion was not associated with outcomes of cervical cancer.

Between 2015 and 2020, the Four-C Study gave rise to 47 articles published in peer-reviewed journals. A list of publications will be available on the website currently under construction.

## Future Plans?

We will further assess outcomes of different management strategies on cervical cancers with specific clinical stages. We will also continue to evaluate the influence of various prognostic factors on the oncological outcomes to guide treatment options, care, and follow-up.

Apart from that, we will also assess the natural history of cervical cancer, including biologic onset, subclinical stage, clinical stage, and outcome as much as possible, through a comprehensive analysis of the detailed information of patients who have not received any treatment or intervention. This will enable us to clarify the yield of different treatments compared with no treatment. The exploration of the natural history of cervical cancer will also contribute to promotion of the early diagnosis and prevention.

We are building a cervical cancer imaging database (33, 39) to collect the Dicom file data of computed tomography (CT)/magnetic resonance imaging (MRI) examinations before treatment of cervical cancer. As of September 10, 2020, CT data of 3042 patients with cervical cancer and MRI data of 2843 patients with cervical cancer have been collected, among which 670 patients have both CT and MRI data. We have manually labeled the tumor boundaries and drawn the contours of the abdominal and pelvic lymph nodes on the collected original imaging. Moreover, we are collecting original pathological samples of patients with cervical cancer. Based on those, we may conduct extensive research on imaging omics and pathology omics of cervical cancer using AI and ML and advanced biological technology.

With digital medicine of obstetrics and gynecology, our team reconstructed a three-dimensional model of female abdominal and pelvic structure based on the computed tomography angiography (CTA) and MRI data sets of participants. We also constructed a digital diagnosis and treatment platform for gynecological and obstetrical diseases. More importantly, we creatively applied digital medicine technology to the preoperative diagnosis, intraoperative guidance, surgical navigation, digital delivery and postoperative evaluation of obstetrical and gynecological diseases.

Finally, our team has committed resources to diagnose difficult miscellaneous diseases using the three-dimensional modeling from the viewpoint of the source of arterial blood supply. In this clinical practice, a pelvic mass of an unknown source can also be successfully identified, which has improved the diagnosis rate of the pelvic mass of an unknown source.

## What Are the Main Strengths and Weaknesses?

This hospital-based cohort has several notable strengths. **Foremost**, it is one of the largest cervical cancer clinical databases to have been established and is likely to be the largest cervical cancer imaging and pathology database worldwide. A sufficiently large sample size allows us to carry out the study restricted to special-interest patient groups (e.g., patients with FIGO stage IB1 to IIA2 cervical cancer) with sufficient statistical power. And this resource may open the door to digital medical research on cervical cancer. **Second**, the cohort included not only cervical cancer patients who were treated with different measures, but also more than 1,400 cervical cancer patients who did not receive any treatment. It means that this cohort is able to provide us a unique opportunity to clarity the progression of cervical cancer and explore the real benefit of different therapeutic methods. **Third**, we have recruited participants in 26 cities of 14 provinces across the mainland China and created a network of 47 sites that include the different types and levels of hospitals. Of all patients (regardless of outpatients and inpatients) in attendance at the 47 sites were enrolled into the cohort. This makes it possible to roughly describe the burden of cervical cancer at different times and in different regions across mainland China, which could potentially inform prevention strategies. **Forth**, detailed information is available on socioeconomic characteristics, imaging, pathology, comorbidities, patient’s management, complications, and other variables, enabling us to conduct comprehensive statistical analyses. Despite difficult working conditions, we have maintained inclusion and follow-up in this large cohort for over 6 years.

One weakness of this project is that some information on clinical outcomes prognoses relies on inpatient medical records, readmission records or outpatient records. As a result, some complications and other related events may be underreported and underestimated. In addition, although all laboratory staff strictly followed the procedure to perform each test, the results of laboratory tests may be affected by the equipment used in different hospitals, while it is really hard to get all 47 hospital to use the same equipment. Finally, several variables, such as those indicating economic status, lifestyle behaviors (smoking status, alcohol assumption and physical activity, etc.), living quality and utilization of medical resource, are not currently collected in the study because they may not be included in routine clinical practice and medical records, from which we obtained the information. We will endeavor to supplement those information during future follow-up, and we plan to include these variables when encountering a new patient with cervical cancer from July 2021.

## Where Can I Find Out More? Can I Get Hold of the Data?

The databases remain the property of all participating centers and will still be managed by the Four-C Study group. Although the databases are not yet freely available in the public domain, potential collaborators are welcomed to contact the corresponding authors, Chun-Lin Chen (e-mail: ccl1@smu.edu.cn) or Chen Mao (e-mail: maochen9@smu.edu.cn). Anyone can submit at least one specific study proposal describing the background, objectives and methods of the study. The databases can be partially transmitted to successful applicants with adequate statistical expertise. Otherwise, the members of the Four-C Study group will cooperatively analyze the data with the applicants. What’s exciting is that we are trying our best to build a public website show the names of all variables and their detailed descriptions. By that moment, anyone may browse the website anytime and anywhere to learn about our project, and users interested in obtaining and using those data could also fill out a Data Use Agreement form downloading from the website to apply for access to the data.

## Data Availability Statement

The original contributions presented in the study are included in the article/supplementary material. Further inquiries can be directed to the corresponding authors.

## Ethics Statement

The studies involving human participants were reviewed and approved by The institutional ethics committee of Nanfang Hospital. Written informed consent for participation was not required for this study in accordance with the national legislation and the institutional requirements.

## Author Contributions

Concept and design: C-LC, CM, J-HL, PL, X-RZ, Z-QL, and L-XS. Data collection and cleaning: PL, P-FL, H-WZ, and B-LC, MJ, LW, SK, and on behalf of Chinese Cervical Cancer Clinical Study collaborative group. Statistical analysis, or interpretation of data: X-RZ, Z-QL, and Z-HL. Drafting of the manuscript: X-RZ, Z-QL, and L-XS. Critical revision of the manuscript for important intellectual content: C-LC, CM, J-HL, PL, H-WZ, and B-LC, MJ, LW, SK. Supervision: C-LC and CM. All authors contributed to the article and approved the submitted version.

## Funding

This study was funded by the National Science and Technology Support Program of China (2014BAI05B03), the National Natural Science Fund of Guangdong (2015A030311024), the Science and Technology Plan of Guangzhou (158100075), Guangdong Medical Science and Technology Research Fund Project (A2020077), Basic and Applied Basic Research Fund of Guangdong Province (2019A1515110337), the Project Supported by Guangdong Province Universities and Colleges Pearl River Scholar Funded Scheme (2019), the Construction of High-level University of Guangdong (G820332010, G618339167 and G618339164), Chinese Postdoctoral Science Foundation (2019M660207), and Nanfang Hospital President Fund (2019C005). The funders played no role in the study design or implementation; data collection, management, analysis or interpretation; manuscript preparation, review or approval or the decision to submit the manuscript for publication.

## Conflict of Interest

The authors declare that the research was conducted in the absence of any commercial or financial relationships that could be construed as a potential conflict of interest.
